# Varying Protein Levels Influence Metabolomics and the Gut Microbiome in Healthy Adult Dogs

**DOI:** 10.3390/toxins12080517

**Published:** 2020-08-12

**Authors:** Eden Ephraim, Chun-Yen Cochrane, Dennis E. Jewell

**Affiliations:** 1 Pet Nutrition Center, Hill’s Pet Nutrition, Inc., Topeka, KS 66617, USA; chun-yen_cochrane@hillspet.com; 2 Department of Grain Science and Industry, Kansas State University, Manhattan, KS 66506, USA; djewell@ksu.edu

**Keywords:** canine, protein levels, plasma, feces, urine, metabolomics, uremic toxins, microbiome, fecal pH, proteolysis

## Abstract

The optimal ranges of protein for healthy adult dogs are not known. This study evaluated the impact of long-term consumption of foods containing low, medium, and high levels of protein on serum, urine, and fecal metabolites, and gut microbiome in beagles. Following maintenance on a prefeed food for 14 days, dogs (15 neutered males, 15 spayed females, aged 2–9 years, mean initial weight 11.3 kg) consumed the low (18.99%, dry matter basis), medium (25.34%), or high (45.77%) protein foods, each for 90 days, in a William’s Latin Square Design sequence. In serum and/or urine, metabolites associated with inflammation (9,10-dihydroxyoctadecanoic acid (DiHOME)), 12,13-DiHOME) and kidney dysfunction (urea, 5-hydroxyindole sulfate, 7-hydroxyindole sulfate, *p*-cresol sulfate) increased with higher protein levels in food, while one-carbon pathway metabolites (betaine, dimethylglycine, sarcosine) decreased. Fecal pH increased with protein consumed, and levels of beneficial indoles and short-chain fatty acids decreased while branched-chain fatty acids increased. Beta diversity of the fecal microbiome was significantly different, with increased abundances of proteolytic bacteria with higher protein food. Feeding dogs a high amount of protein leads to a shift to proteolytic gut bacteria, higher fecal pH, and is associated with increased levels of metabolites linked with inflammation and kidney dysfunction.

## 1. Introduction

The optimal range of protein required for healthy adult dogs has not been demonstrated. The latest Association of American Feed Control Officials (AAFCO) Dog Food Nutrient Profile recommends a minimum crude protein concentration of 18.0% on a dry matter basis for a 4000 kcal/kg adult maintenance food [1]. The AAFCO profile also states minimums for essential amino acids; however, there are no optimum concentrations and no upper limits suggested. Commercial dog foods contain differing amounts of protein, ranging from 18% to over 60%, with equally divergent amino acid concentrations.

While some studies have tested the effects of meeting the dietary amino acid recommendations while supplying high protein levels on canine health on the microbiome and metabolome [2,3,4], few have evaluated a range of protein levels [5]. As in humans, dog foods high in protein have been explored in recent years, particularly for weight loss [6,7,8,9]. However, the health effects of a high protein diet have not been fully established. In fact, there is some evidence that consumption of high meat food without the addition of fiber favors proteolytic catabolism over the more beneficial saccharolysis [10,11]. Additionally, increases in fecal pH and metabolites such as branched-chain fatty acids (BCFAs) and indole sulfates, which originate from microbial fermentation of amino acids such as tryptophan, have been observed in dogs fed high protein foods [4,5,10,11,12].

The microbiome and the postbiotics derived from microbial metabolic activity are known to influence host health, though the extent of their causality on health largely remains to be established [13,14]. Metabolites present in the bloodstream can be derived from microbiome-mediated metabolism in the colonic lumen [15,16]. Thus, characterization of metabolites from the blood as well as those from the feces can provide insight into host health. Moreover, canine microbiome data may provide insight into microbiota in humans [7], so investigation into the effect of high protein diets is of great interest.

In this study, the effect of a 90-day consumption of varying levels of protein (low (18.99%), medium (25.34%), and high (45.77%) on a dry matter basis) in healthy adult dogs was examined in order to assess various indicators of health and metabolism. Effects on body weight, metabolites in serum, urine, and feces, and composition of the gut microbiome were evaluated.

## 2. Results

### 2.1. Proximate Analysis and Digestibility of Test Foods

Three foods with different protein levels, each of which was consumed for a 90-day period in a Williams Latin Square design, were produced by varying the levels of dried chicken and soybean protein, with final dry matter protein levels of 18.99%, 25.34%, and 45.77%. Proximate analyses showed slightly higher levels of ash and crude fat in the food with the highest levels of protein (Table S1). Digestibilities were largely similar across the three food types, with the exception of the digestibility of apparent protein and fiber.

### 2.2. Study Animals and Body Weight

Thirty healthy spayed/neutered beagle dogs, 15 male and 15 female, were included in this study. The mean ± standard deviation age was 5.7 ± 1.5 years and initial body weight was 11.3 ± 2.3 kg. No adverse events were reported during the study period, and no dogs were removed from the study.

There was no significant difference in the intake of foods of varying protein levels (kcal or dry matter; *p* = 0.12). Likewise, consumption of differing levels of protein did not significantly affect body weight (*p* = 0.64).

### 2.3. Serum Metabolites

**Figure 1:**
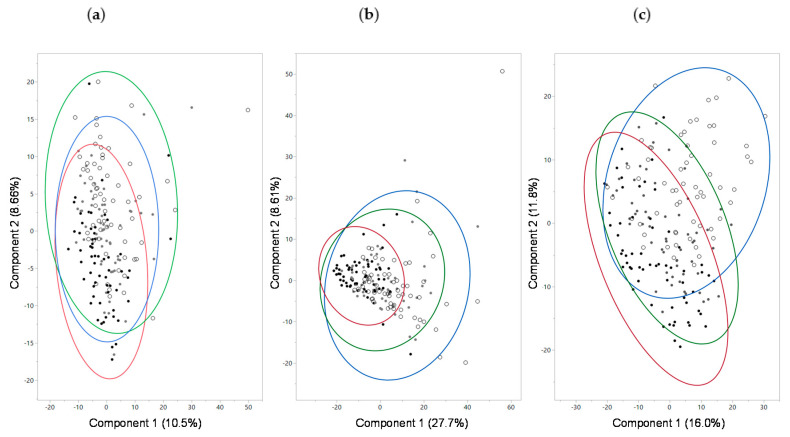
Principal components (PC) analysis for metabolomics data from (**a**) serum, (**b**) urine, and (**c**) feces from dogs fed food containing high (black dots), medium (gray dots), or low (white dots) levels of protein. Red, green, and blue circles indicate 95% confidence intervals for the high, medium, and low protein foods, respectively. For (**a**), effects on PC1 were significant between medium and high protein foods (*p* = 0.0003) and low and medium protein foods (*p* = 0.0008); effects on PC2 were significant between medium and high protein foods (*p* < 0.0001) and low and medium protein foods (*p* < 0.0001). For (**b**), effects on PC1 were significant between low and high protein foods (*p* < 0.0001); effects on PC2 were significant between low and high protein foods (*p* = 0.03). For (**c**), effects on PC1 were significant between low and high protein foods (*p* < 0.0001); effects on PC2 were significant between low and high protein foods (*p* < 0.0001).

Metabolomic analysis of serum from samples 45 and 90 days identified 620 metabolites. Principal component analysis (PCA) of the serum metabolomics did not show complete separation; however, differences were significant on principal component 1 (PC1) between consumption of both medium and high protein foods (*p* = 0.0003) and between low and medium protein foods (*p* = 0.0008; Figure 1a). Similarly, there was a significant effect on PC2 between both medium and high protein foods (*p* < 0.0001) and between low and medium protein foods (*p* < 0.0001). Eigenvectors with their corresponding eigenvalues for PC1 and PC2 are shown in Table S2. Since creatinine, an indicator of kidney dysfunction, appeared as an eigenvector for the PCA, serum chemistry (separate from the metabolomics analysis) results were examined. Mean (standard error (SE)) values of serum creatinine after consumption of the high, medium, and low protein foods were 0.81 (0.02), 0.78, (0.02), and 0.78 (0.02) mg/dL, respectively, and did not significantly differ among the foods. Levels of creatinine were only significantly different between the high protein food and the prefeed (i.e., baseline: 0.74 (0.04), *p* = 0.0084). Other results of the serum chemistry analysis showed that dogs had significantly increased level of triglycerides on the high protein food 78.9 (5.3) compared with the medium 66.8 (5.3; *p* = 0.03) or the low 69.1 (5.0; *p* = 0.01) protein foods. None of the other serum chemistry results were significantly different between treatments.

**Figure 2:**
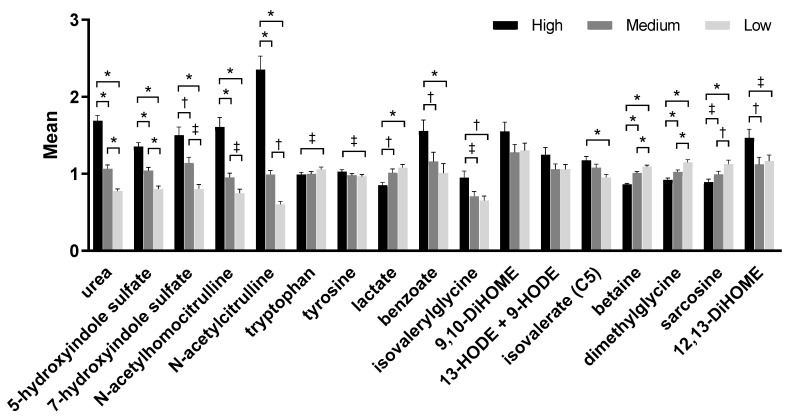
Selected metabolites from metabolomic analyses from serum samples of dogs fed the high, medium, and low protein foods. Data were rescaled to a median value of 1 and are presented as group means and standard errors. Significance was determined using one-way ANOVA followed by a post hoc Tukey’s test. * *p* ≤ 0.001; ^†^ *p* ≤ 0.01; ^‡^ *p* ≤ 0.05. DiHOME, dihydroxyoctadecanoic acid; HODE, hydroxyoctadecadienoic acid.

Serum metabolites such as indole sulfates, tryptophan, lactate, benzoate, and tyrosine were among the eigenvectors that led to the significant difference of the serum principal components by treatment. Levels of uremic toxins such as urea, 5-hydroxyindole sulfate, and 7-hydroxyindole sulfate increased significantly as food protein levels increased (Figure 2, Table S3). Metabolites of one-carbon metabolism including sarcosine, dimethylglycine, and betaine also contributed to these differences significantly; levels of all the three metabolites decreased significantly with increased consumption of protein. Levels of a microbial lipid associated with inflammation, 12,13-dihydroxyoctadecanoic acid (12,13-DiHOME), were significantly higher after consumption of the high protein food compared to both the medium (*p* = 0.008) and the low protein (*p* = 0.023) foods. A similar metabolite, 9,10-DiHOME, also tended to be higher after consumption of the high protein food but did not reach significance (*p* = 0.057 vs. the medium protein food).

### 2.4. Urine Metabolites

**Figure 3:**
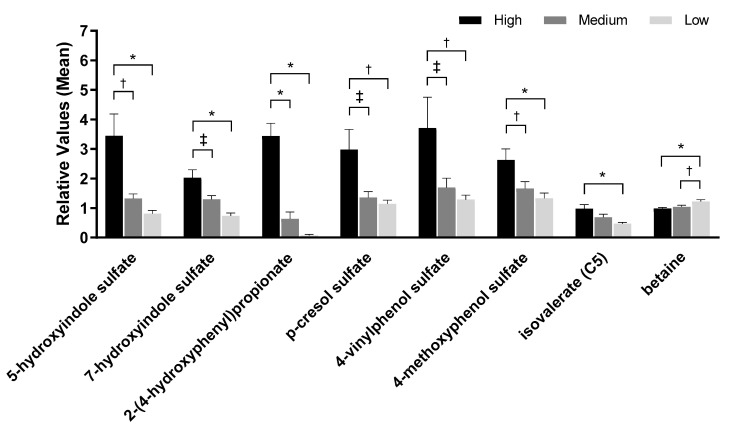
Metabolite analyses from urine samples of dogs fed the high, medium, and low protein foods. Data were rescaled to a median value of 1 and are presented as group means and standard errors. Significance was determined using one-way ANOVA followed by a post hoc Tukey’s test. * *p* ≤ 0.001; ^†^ *p* ≤ 0.01; ^‡^ *p* ≤ 0.05.

Metabolomic analysis of urine from samples 45 and 90 days identified 627 metabolites. PCA of the urine metabolomics data showed no complete separation by treatment; however, there was a significant effect on PC1 between consumption of low and high protein foods (*p* < 0.0001) but not between medium and high protein foods (*p* = 0.27; Figure 1b). A significant effect on PC2 was seen between low and high protein foods (*p* = 0.03) but not between medium and high protein foods (*p* = 0.85). One of the eigenvectors that contributed to the significance was betaine. Consistent with the serum metabolome findings, the level of betaine in urine decreased with increasing levels of protein consumption. In addition, the branched-chain amino acid isovalerate increased with increased protein consumption. Metabolites linked to kidney disease and inflammation (4-methoxyphenol sulfate, 4-vinylphenol sulfate, 5-hydroxyindole sulfate, 7-hydroxyindole sulfate, 2-(4-hydroxyphenyl)propionate, and *p*-cresol sulfate) were also significantly higher in urine with high protein consumption (Figure 3, Table S3).

### 2.5. Fecal pH and Metabolites

**Figure 4:**
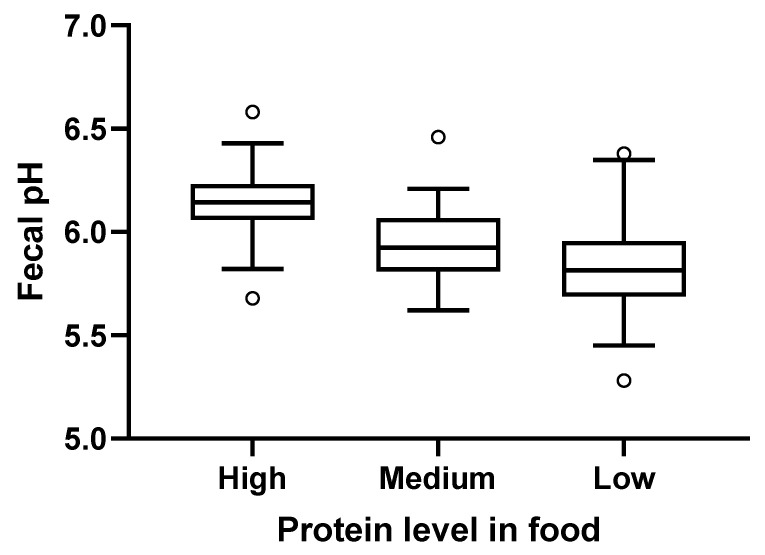
Fecal pH levels from dogs fed the high, medium, or low protein foods. Lines within the boxes indicate the median pH; tops and bottoms of the boxes represent the upper and lower quartiles, respectively. Circles represent the minimum and maximum values.

Fecal pH increased with the levels of protein in the consumed foods, with fecal pH (mean (SE)) highest after consumption of the high protein food (6.2 (0.02)) compared with the medium (5.9 (0.02)) and the low protein (5.8 (0.03)) foods (Figure 4). Stool scores were satisfactory, with mean (SE) values of 4.5 (0.08) for low, 4.7 (0.06) for medium, and 4.8 (0.06) for the high protein foods.

**Figure 5:**
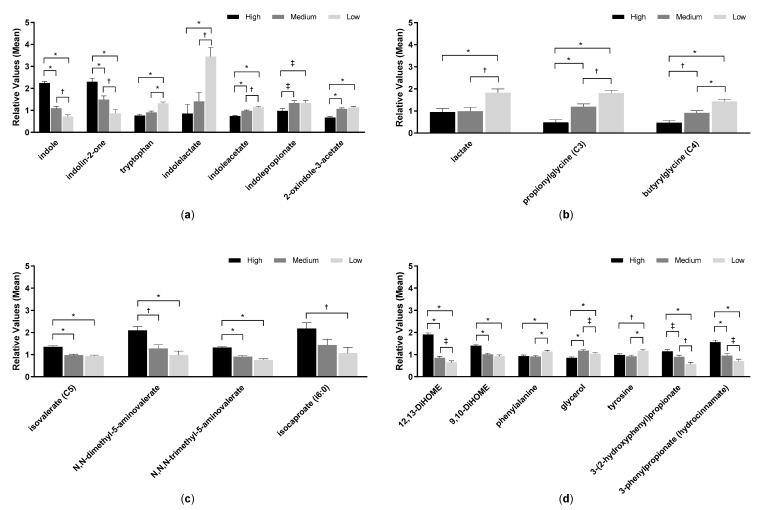
Metabolite analyses from fecal samples of dogs fed the high, medium, and low protein foods; (**a**) indoles, (**b**) short-chain fatty acids, (**c**) branched-chain fatty acids, and (**d**) other metabolites. Data were rescaled to a median value of 1 and are presented as group means and standard errors. Significance was determined using one-way ANOVA followed by a post hoc Tukey’s test. * *p* ≤ 0.001; ^†^ *p* ≤ 0.01; ^‡^ *p* ≤ 0.05. Levels of short-chain fatty acids (SCFAs), which generally confer beneficial effects on health, decreased with increased protein consumption (Figure 5b, Table S3), while BCFAs increased with increased protein consumption (Figure 5c, Table S3).

Metabolomic analysis of feces from samples 45 and 90 days identified 702 metabolites. PCA of fecal metabolites showed no complete separation by treatment; however, PC1 was significantly different between consumption of low and high protein foods (*p* < 0.0001) but not between medium and high protein foods (*p* = 0.23; Figure 1c). Similarly, treatment had a significant effect on PC2 between low and high protein foods (*p* < 0.0001) but not between medium and high protein foods (*p* = 0.23). Levels of amino acids, indoles, DiHOME, and benzoate metabolites were among the top eigenvectors that led to the significant differences. Fecal levels of indole and indolin-2-one significantly increased with protein consumption, while levels of beneficial indoles (indolelactate, indoleacetate, indolepropionate, and 2-oxindole-3-acetate) significantly decreased with increasing levels of protein consumed (Figure 5a, Table S3). Tryptophan, which is the precursor amino acid of indoles, increased with decreased consumption of protein.

Further, inflammation-associated microbial metabolites such as 12,13-DiHOME and 9,10-DiHOME significantly increased after consumption of the high protein food. Benzoate metabolites such as 3-phenylpropionate and 3-(2-hydroxyphenyl)propionate also increased with increased protein intake (Figure 5d, Table S3).

### 2.6. Fecal Microbiome

Ninety-one operation taxonomic units (OTUs) passed the filtration criteria to be considered for statistical analysis. The Shannon and inverse Simpson alpha diversity measures were similar among the fecal microbiomes following consumption of the three treatment foods (Figure S1a). Beta diversity, which indicates intragroup microbial dissimilarity, was significantly different among feces from dogs that consumed the three foods with varying protein levels (FDR-corrected *p* < 0.01 for all pairwise comparisons), although neither principal coordinate analysis PCoA (Figure S1b) nor a heatmap of the relative abundance data (Figure S1c) showed any obvious patterns among the three treatment foods.

**Figure 6:**
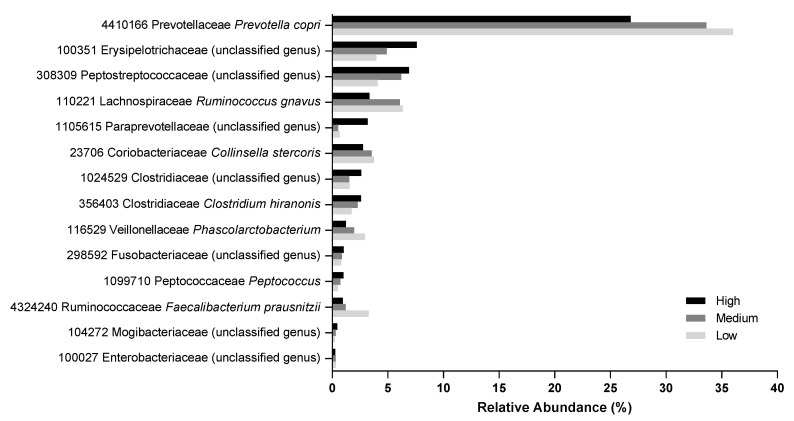
Relative abundance (percentage) of operational taxonomic units (OTU) that showed significant changes in fecal samples of dogs fed the high, medium, and low protein foods. Operational taxonomic unit number, family, and genus are shown (species also included where available).

There were 39 OTUs that had significant difference in abundance between at least two food types (Table S4). Several OTUs were significantly decreased in the feces of dogs after they were fed the high protein food relative to after the medium or low protein foods. These included those from the genera *Prevotella* (OTU 4410166), *Ruminococcus* (OTU 110221), *Collinsella* (OTU 23706), *Phascolarctobacterium* (OTU 116529), and *Faecalibacterium* (OTU 4324240; Figure 6). Conversely, the relative abundance of unknown genera in the families Erysipelotrichaceae (OTU 100351), Peptostreptococcaceae (OTU 308309), Paraprevotellaceae (OTU 1105615), and Clostridiaceae (OTU 1024529) were increased with the high protein food (Figure 6). The metabolic function predicted by Phylogenetic Investigation of Communities by Reconstruction of Unobserved States (PICRUSt) indicated there were significant differences (FDR corrected *p* < 0.05) in the phenylalanine, tyrosine, propionate, tryptophan, butyrate, benzoate, and arginine pathways among the three treatments (Table S5).

## 3. Discussion

In this study, the increasing apparent digestibility of protein is easily seen to be an effect of the integumental nitrogen loss being diluted out by the increasing amount of ileal bypass protein. The true protein digestibility shows that the foods were similar in protein digestibility, and therefore protein presented to the large intestine for fermentation was directly responsive to dietary protein concentration. Overall, metabolites associated with kidney disease and inflammation increased with levels of protein consumption in healthy dogs. Our results using highly digestible protein show that there can be a negative effect in dogs with increasing dietary protein, which results in increased protein substrate available to the microbiota. It is likely that foods with lower protein digestibility even at the same protein concentration would elicit the same response because of the increase in protein bypassed to the colon.

It has been previously shown that a higher concentration of circulating urea is expected with increased protein intake [17]. However, an increase in the microbial uremic toxins such as indole sulfates originating from colonic tryptophan fermentation is associated with kidney disease and inflammation [18]. In our study, levels of indole sulfates and *p*-cresol in urine increased with increasing protein consumption. *P*-cresol sulfate is one of the gut-derived uremic toxins derived from putrefaction of undigested dietary protein [19]. In fact, both indole sulfates and *p-*cresol sulfate stimulate significant cellular inflammation [20].

The levels of the one-carbon metabolism pathway metabolites betaine, dimethylglycine, and sarcosine all significantly decreased in serum as protein consumption increased, and betaine also significantly decreased in urine. Betaine has several functions including serving as an osmoprotectant, anti-inflammatory, antioxidant, and methyl donor [21]. In the one-carbon metabolism pathway, betaine donates its methyl group to the uremic toxin homocysteine, which is converted to methionine as betaine becomes dimethylglycine. Betaine-enriched foods are also reported to modulate the gut microbiome by increasing the abundance of beneficial bacteria [22]. The significant reductions of betaine and its metabolites with increased protein intake suggest a surprising shift away from the one-carbon metabolism pathway and may have negative physiological consequences with long-term consumption of high protein foods.

A greater number of published studies have reported on the fecal metabolome (and microbiome) than on serum or urine metabolites in response to foods varying in protein concentration. Results from the present study were largely consistent with prior studies on the effect of higher protein levels in dog food, though nearly all of the latter examined only one or two levels of protein and usually compared against a carbohydrate-based food with a different ingredient rather than different protein to carbohydrate ratios. This suggests a further avenue of study in which an increased protein intake is evaluated in the presence of changing microbial cofactors such as resistant starch or other polysaccharides available for saccharolytic bacteria metabolism.

Fecal pH is an indicator of saccharolysis and proteolysis activities in gut microbes, with higher pH corresponding to proteolytic metabolism. Fecal pH increased with protein consumed, consistent with other studies that have shown higher pH in protein-rich versus carbohydrate-rich foods [4,5,10,11]. Likewise, lower levels of SCFAs and higher levels of BCFAs are associated with proteolysis and gut microbe-mediated putrefaction, and the directionality with consumption of high protein food also corroborates prior work [4,11]. It is widely documented that SCFAs (which result from saccharolytic fermentation) have several benefits to the host, in particular due to their inhibitory effect on pathogenic microorganisms by lowering the luminal pH, their anti-inflammatory properties, and by being an important source of energy for colonocytes [23]. In contrast, other reports show that BCFAs have beneficial properties, including suppression of pro-inflammatory markers in human intestinal epithelial cells [24], cytotoxicity to cultured breast cancer cells [25], and reduction of necrotizing colitis in neonatal rats [26]. Since microbial-generated BCFAs are also generated from the fermentation of branched-chain amino acids [27], it may follow that BCFA levels would increase after high protein consumption. Indeed, with increasing protein levels in the food, fecal levels of lactate and the glycine conjugates of SCFAs decreased and levels of isovalerate and its derivatives increased. (SCFAs alone are too volatile to be detected in the metabolomic screen.)

Consistent with prior results, components of the tryptophan pathway increased (indole, indolin-2-one) or decreased (tryptophan, 2-oxindole-3-acetate, indoleacetate, indolelactate, indolepropionate) in feces with higher protein consumption [10]. Those that decreased are known to be beneficial indoles that are important to maintain gut barrier integrity [28]. Other metabolites that have been associated with inflammation were detected in dogs that consumed the high protein food such as the proinflammatory fatty acids 9,10-DiHOME and 12,13-DiHOME [29], which were significantly increased in feces from dogs on the high protein food compared to the low or medium protein food.

Some gut bacteria were of lower (genera *Prevotella* and *Faecalibacterium*) or higher (unknown genera in the families Clostridiaceae and Fusobacteriaceae) abundance with higher protein consumption in agreement with prior work [3,8]. Shifts in the abundances of these bacteria correlated with differences in several metabolic pathways, including those of phenylalanine, tyrosine, propionate, tryptophan, butyrate, benzoate, and arginine. Many of the OTUs that were decreased as food protein levels increased are saccharolytic, including *Prevotella copri* [30] and *Ruminococcus gnavus* [31]. Others that were of higher abundance with greater food protein levels include unknown genera from the families Erysipelotrichaceae, Peptostreptococcaceae, and Clostridiaceae and genus *Peptococcus*, which are implicated in inflammation [32] and/or proteolytic metabolism [33].

Several of the bacteria identified in this study point to a benefit of consumption of lower protein levels. Here, the relative abundance of *Faecalibacterium prausnitzii* was increased with consumption of the low protein food. This bacterium produces the beneficial SCFA butyrate and has anti-inflammatory properties [34] and is reduced in humans with Crohn disease [35] and irritable bowel syndrome [36]. In addition, *Collinsella*, which were of lower abundance with the high protein food in this study, are also of lower abundance in dogs with acute diarrhea or inflammatory bowel disease compared with healthy dogs [37].

In contrast, some of the bacteria identified as responding to protein levels in food in this study have been associated with both beneficial and detrimental effects. For example, *P. copri* is saccharolytic but has been shown to induce insulin resistance and increase levels of circulating BCFAs [38] and is implicated in the pathogenesis of rheumatoid arthritis [39]. In fact, it appears that the type of *P. copri* strain and its associated metabolic activity are influenced by host diet [40]. In addition, *R. gnavus*, the abundance of which decreased with increasing protein levels in food in this study, produces a peptide with antimicrobial activity against the pathogen *Clostridium perfringens* [41]. However, the abundance of *R. gnavus* is increased in humans with inflammatory bowel conditions and produces an inflammatory polysaccharide [42,43]. Furthermore, it is important to note that it is not necessarily the composition of the microbiome but rather its metabolic capacity that may have the largest effect on the host. Altogether, these data highlight the need for further research into the interplay among the microbiome, the metabolome, and dietary effects on the host organism.

Limitations of this study include absence of metagenomic data to determine changes in the abundance of specific microbial strains and their functions with varying protein intake. Further, measurements of SCFAs in feces would provide additional information associated with changes in the microbiome and fecal pH. However, the glycine conjugates of SCFAs were detectable in the metabolic screen. In addition, this study could not differentiate between increasing concentration of the protein sources (chicken and soy) and increases in the total protein intake. Further research is needed to evaluate the effects of different protein sources on the metabolome and microbiome. Finally, it is important to note that the results of this study demonstrate an effect of increased dietary protein on metabolites and microbiota that are indicators of ill health, but that ill health of the dogs was not observed.

Prior studies have shown that there may be some value in high protein diets in order to promote weight loss in obese dogs [6,7,8]. In this study, the body mass of healthy dogs of normal weight was not affected by the protein levels in food. This was as expected because not only were the amino acids significantly above the minimum AAFCO recommendations, they also exceeded the levels in a prior canine nutrition study [44]. For example, the AAFCO minimum tryptophan concentration is 40 mg/100 kcal) metabolizable energy. The minimum tested in the prior study (with all levels shown to be adequate) was 50.5, and the minimum level in the present study was 57.6 mg tryptophan/100 kcal. All other essential amino acids in this study exceeded the AAFCO minimums by greater than the amount exceeded by tryptophan.

The results here indicate that consumption of high protein food over the long-term increases in metabolites associated with kidney dysfunction, inflammation, and proteolysis. The consequences of these changes in the overall canine health should be confirmed and further characterized with future work.

## 4. Materials and Methods

### 4.1. Study Foods

Protein levels in the high, medium, and low protein foods varied by the amount of dried chicken (25.4%, 17.0%, and 11.0%, respectively) and soybean protein isolate (19.6%, 3.0%, and 0%). Macronutrient composition and amino acid compositions of foods was determined by a commercial laboratory (Eurofins Scientific, Inc., Des Moines, IA, USA), and all were completed using the respective Association of Analytical Communities (AOAC) methods [45]. All foods met the AAFCO maintenance nutrition requirements, while exceeding the minimum amino acid requirements by over 20%. The medium protein food represented the average protein level present in commercially available dog foods, whereas the low protein food contained the minimum AAFCO-recommended concentration for healthy adult dogs. The high protein food was designed to contain the highest protein concentration possible for a dry kibble. Formulations and proximate analysis were carried out as previously described [10]. All foods were in dry form, and true and apparent digestibility assays were performed as previously described [46].

### 4.2. Animals and Experimental Design

Thirty healthy beagle dogs of 2–9 years of age, owned by Hill’s Pet Nutrition, Inc., were included. Dogs with diseases, including compromised kidney and intestinal function (e.g., inflammatory bowel disease, colitis), or food allergy and those that received antibiotics or vaccines less than a month before study start were excluded. Dogs were to be removed from the study if, in the opinion of the colony veterinarian, they would benefit from removal. All dogs were housed in pairs in spacious rooms with regular access to natural light at the Pet Nutrition Center. Exercise was provided daily, and dogs had regular opportunities for socialization.

All work was approved by the Hill’s Institutional Animal Care and Use Committee (IACUC; #CP747) and Animal Welfare Committee (Date: 16 February 2017). This study complied with the guide for the care and use of laboratory animals from the US National Research Council [47].

**Figure 7:**
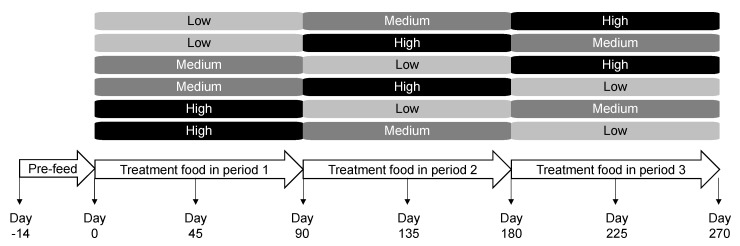
Study design and timeline in which dogs consumed the low, medium, and high protein foods in a Williams Latin Square sequence.

For 14 days, all dogs were maintained on Hill’s^®^ Science Diet^®^ Canine Maintenance^®^ containing 230 g/kg crude protein (major protein source: chicken), 134.9 g/kg crude fat, 49.9 g/kg ash, 180 g/kg crude fiber, and 86.0 g/kg moisture. Dogs were then randomly assigned to one of the six different groups of five dogs each (Figure 7). Age and gender were balanced across the groups. Each group consumed one of the low, medium, or high protein foods for 90 days followed by the other treatment foods in a distinct sequence following the Williams Latin Square Design [48]. Dogs were fed from electronic feeders, where fresh food was offered once a day with amounts calculated to maintain body weight. Blood, urine, and fecal samples were collected at the end of the pre-feed period as well as approximately on days 45 and 90 of each treatment period. Body weight and levels of metabolites and microbial composition were assessed at the end of each 90-day feeding period. All dogs were assessed to be healthy at the end of the study.

### 4.3. Stool Scoring and Fecal Sample Processing

Stool quality was assessed on a scale ranging from 1 (non-solid form) to 5 (>80% firm) [49]. Feces were processed for metabolome and microbiome analysis as previously described [10].

### 4.4. Metabolite Analyses

Metabolomics was performed on serum, urine, and fecal samples by Metabolon (Morrisville, NC, USA) as previously described [10]. Mean units for metabolites are presented as relative levels after scaling for a median of 1.

Serum chemistry was performed to measure levels of triglycerides, creatinine, albumin, and cholesterol according to Hall et al. [50].

### 4.5. Fecal Microbiome Analysis and Bioinformatics Processing

The fecal microbiome analysis was performed as previously described [10], except that total DNA was extracted from frozen feces samples using the Qiagen MagAttract Power Microbiome DNA/RNA EP DNA isolation kit (Qiagen Cat. No. ID:27500-4-EP, Germantown, MD, USA) optimized for use with the Eppendorf epMotion 5075 TMX platform (Eppendorf, AG, Hamburg, Germany). In brief, PCR amplification spanned the V3–V4 hypervariable regions of the 16S rRNA gene, amplicon sequencing was performed using the Illumina library preparation protocol (15044223 Rev. A) and sequences were de-multiplexed to obtain FASTQ Files. Sequences with a quality score (Q30) above 80% were selected. FASTQ files were then pre-processed into contigs from pairs of reads, chimeras were removed, and Mothur software (University of Michigan, Ann Arbor, MI, USA) was used to obtain bacterial taxonomic classification per the GreenGenes reference taxonomy at the genus level. OTUs were identified based on taxonomic hierarchy and were further processed using the PICRUSt protocol to correct for copy numbers of the 16S genes in their respective taxa. Samples with <5000 reads were removed from the analysis. Sequences were deposited in the NCBI Sequence Read Archive under Accession No. PRJNA649283.4.6.

### 4.6. Statistical Analysis

Analysis of variance followed by Tukey’s post hoc test was completed using treatment food and animal identification so that intra-individual variation was evaluated in the analysis of metabolomics data. PCA and identification of eigenvectors on the principal components were performed using the JMP Pro software (JMP, Cary, NC, USA). In all analyses, statistical significance was considered as *p* ≤ 0.05. For the fecal microbiome data, the 16S copy number-corrected OTU counts and PICRUSt-predicted functional data were first filtered by prevalence. Only those that passed the 80% prevalence in at least one of the food treatment groups were considered for statistical analysis. The counts of individual OTUs were analyzed by negative binomial mixed models using the R BhGLM package [51]; diet treatment, phase day, and sequence effects were considered as fixed, and the animal identification as random. Data from day 45 and day 90 samples were analyzed using mixed model analysis; no temporal differences were identified, so the data from both time points were used herein.

Alpha diversity was calculated on genus-level count data using the R vegan package [52] and was evaluated as the Shannon index and the inverse of the Simpson index using unfiltered OTU data. A principal coordinate analysis was performed on the Manhattan distance of the relative abundance of the 91 OTUs that passed the criteria for statistical analysis. PERMANOVA based on the Manhattan distance of the relative abundances was used to compare beta diversity and pathway functional compositions between diet treatments; the analysis was carried out using the adonis function in the R vegan package [52]. The PERMANOVA and all *p*-values were adjusted for false discovery rate (FDR) by the Benjamini and Hochberg procedure [53]. A heatmap was generated based on the day 90 relative abundance data of the 91 OTUs that passed the criteria for statistical analysis.

## Supplementary Materials

The following are available online at https://www.mdpi.com/2072-6651/12/8/517/s1, Figure S1: Alpha (a) and beta (b) diversity analysis and heatmap of OTU relative abundances (c) from fecal samples from dogs fed the high, medium, and high protein foods, Table S1: Formulations, proximate analyses, and digestibility of the three test foods, Table S2: Eigenvectors and their corresponding eigenvalues on principal components 1 and 2 for the serum, urine, and fecal metabolomic analysis, Table S3: Full data for the metabolomic analyses from the serum, urine, and fecal samples from dogs fed the high, medium, and low protein foods, Table S4: Microbiome data from fecal samples from dogs fed the high, medium, and low protein foods, Table S5: Microbiome PICRUSt data from fecal samples from dogs fed the high, medium, and low protein foods.
