# Supplementary material for: Varying Protein Levels Influence Metabolomics and the Gut Microbiome in Healthy Adult Dogs
Source: Toxins (Basel). 2020 Aug 12;12(8):517. doi: 10.3390/toxins12080517 (PMC7472411; doi:10.3390/toxins12080517)
Supplement: Supplementary file 1 [file toxins-12-00517-s001.zip › Canine ptn levels_Figure S1 and Table S1.docx]

Supplementary Materials: Varying Protein Levels Influence Metabolomics and the Gut Microbiome in Healthy Adult Dogs

Eden Ephraim, Chun-Yen Cochrane and Dennis E. Jewell

| 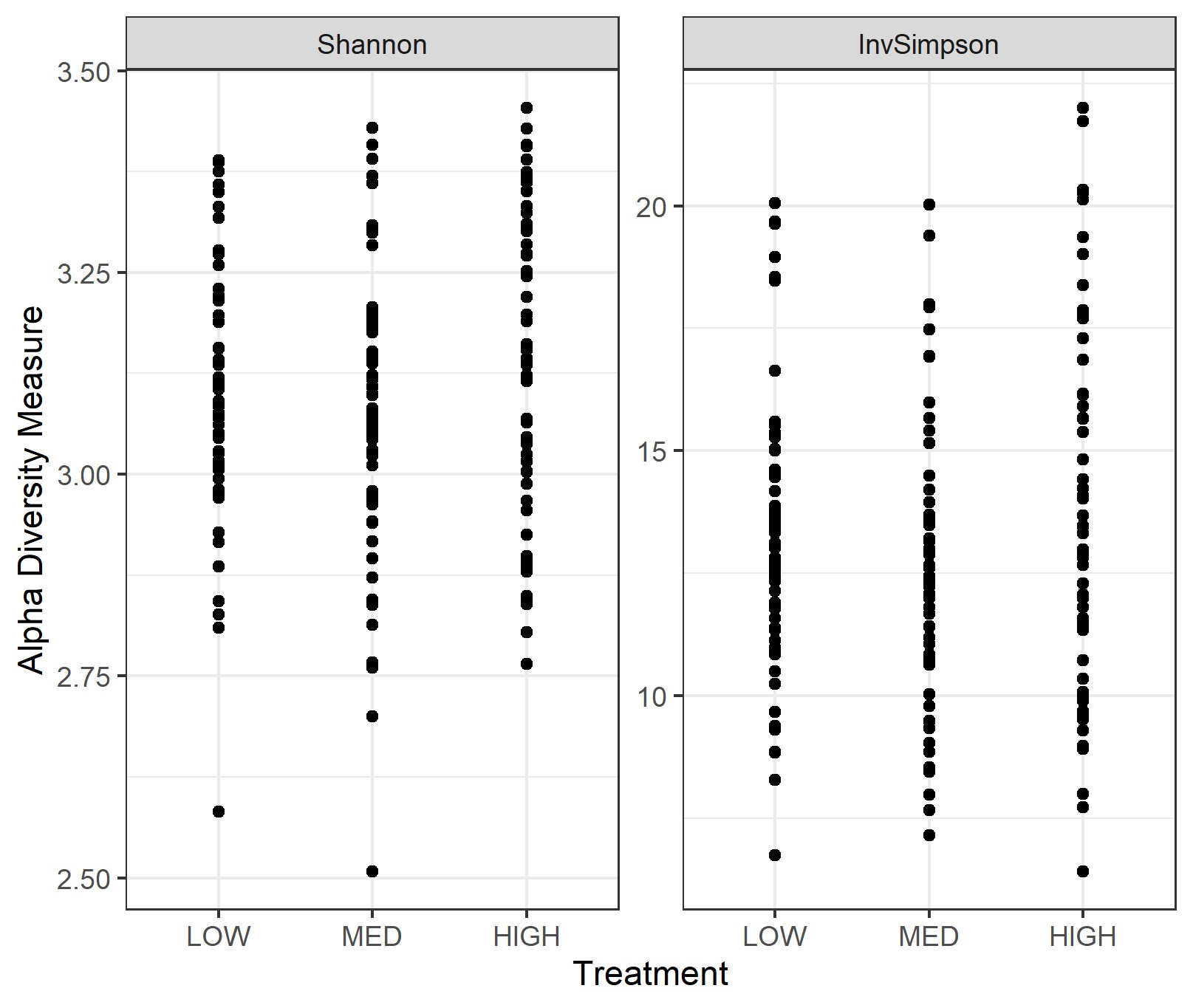 |
| --- |
| (a) |
| 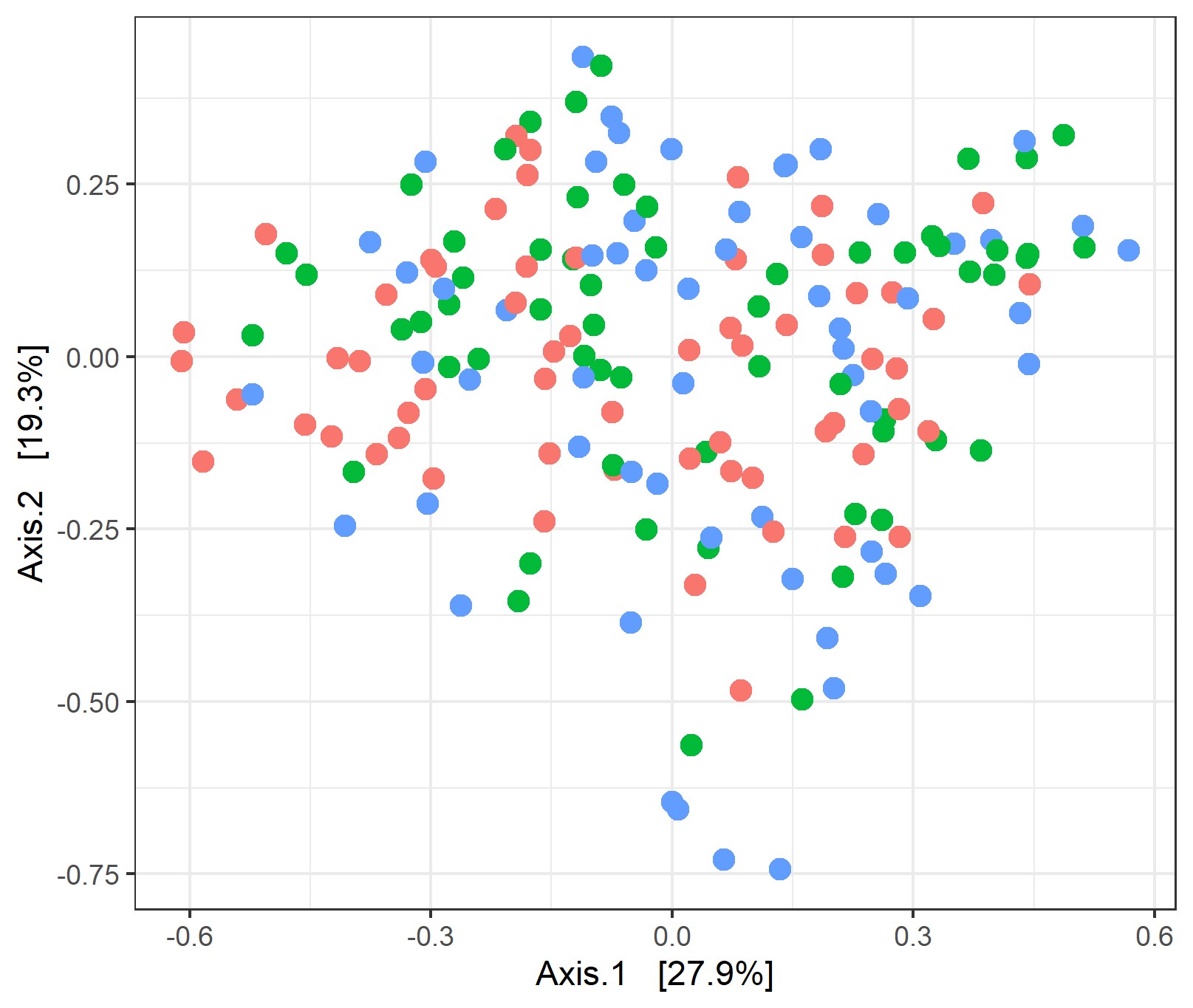 |
| (b) |
| 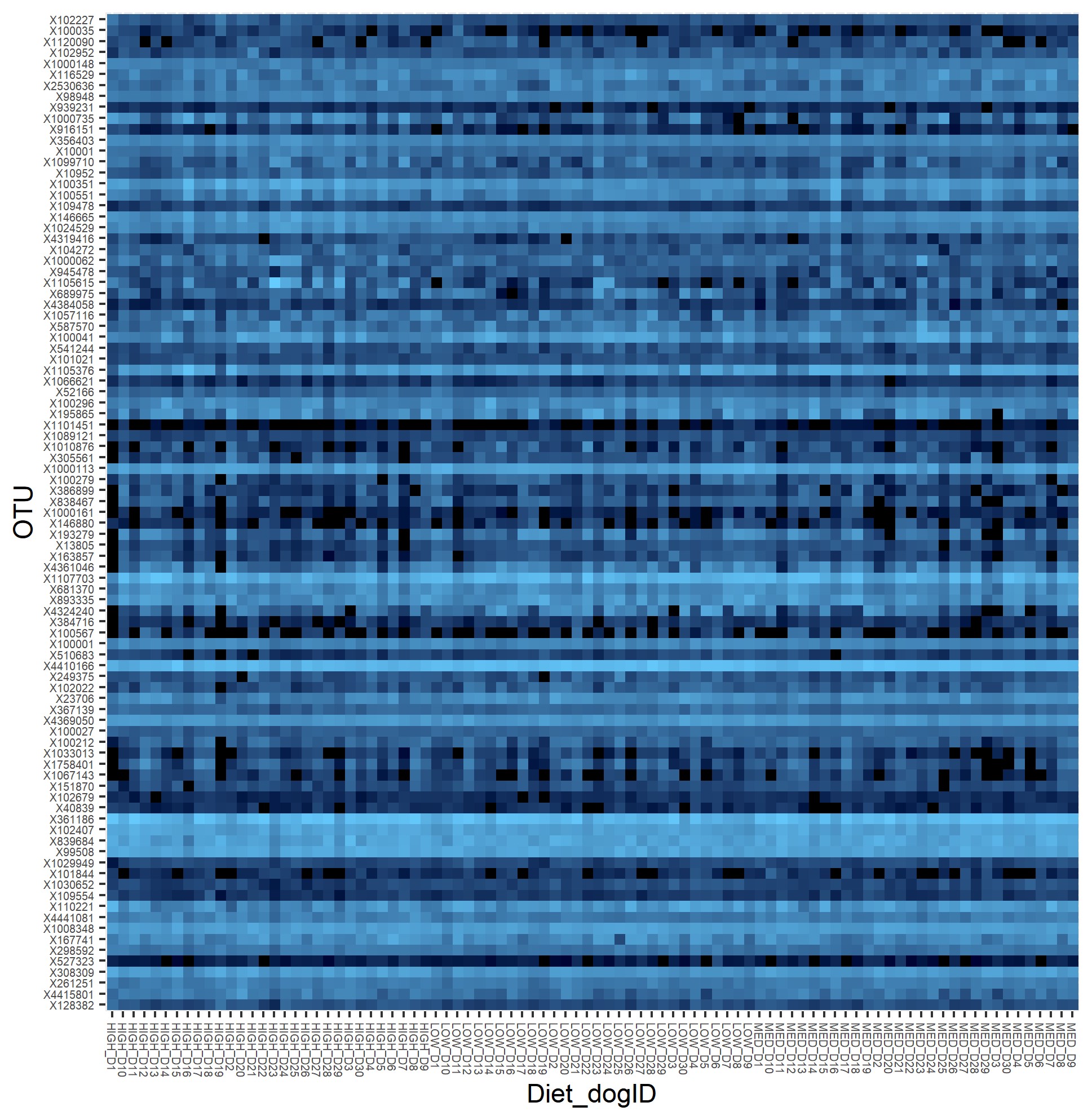 |
| 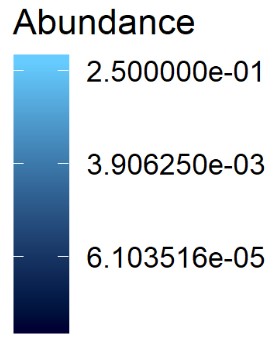 |
| (c) |

**Figure S1.** (**a**) Alpha diversity plots for the Shannon and inverse Simpson indices, (**b**) principal coordinate analysis of the relative abundances of operational taxonomic units (OTUs) from dogs fed the high (blue), medium (green), and low (red) protein foods, and (**c**) heatmap of the relative abundances of OTUs.

**Table S1.** Proximate analyses and digestibility of the three test foods.

|  | **Protein Level** | | |
| --- | --- | --- | --- |
|  | **High** | **Medium** | **Low** |
| **Proximate Analysis, g/kg** |  |  |  |
| Ash | 47.8 | 39.3 | 37.8 |
| Crude fat | 92.7 | 79.8 | 71.5 |
| Crude fiber | 6.0 | 9.0 | 10.0 |
| Neutral detergent fiber (NDF) | 30 | 90 | 46 |
| Crude protein | 420 | 232 | 174 |
| Moisture | 73.9 | 81.6 | 83.0 |
| **Digestibility** |  |  |  |
| Apparent dry matter digestibility, % | 89.8 | 88.7 | 90.4 |
| Apparent protein digestibility, % | 93.1 | 87.1 | 86.7 |
| True protein digestibility, % | 95.9 | 93.5 | 94.5 |
| Apparent fat digestibility, % | 90.4 | 88.9 | 88.6 |
| Apparent fiber digestibility, % | 8.6 | 2.5 | 2.7 |
| Apparent carbohydrate digestibility, % | 94 | 95 | 96 |
| Apparent energy digestibility, % | 92.1 | 90.4 | 91.7 |
| Diet gross energy, kcal/kg | 4760 | 4490 | 4380 |
| Food metabolizable energy, kcal/kg^a^ | 3900 | 3810 | 3820 |
| Nitrogen-free extract calories, % | 35.9 | 57.4 | 65.1 |
| Protein calories, % | 43.5 | 23.4 | 18.2 |
| Fat calories, % | 20.6 | 19.2 | 16.7 |

^a^Tested by the Association of American Feed Control Officials. IU, international units; IU/g, international units per gram; IU/kg, international units per kilogram; ppm, parts per million.
